# Exocytosis through the Lens

**DOI:** 10.3389/fendo.2013.00147

**Published:** 2013-10-17

**Authors:** Alicja Graczyk, Colin Rickman

**Affiliations:** ^1^Institute of Biological Chemistry, Biophysics and Bioengineering, School of Engineering and Physical Sciences, Heriot-Watt University, Edinburgh, UK

**Keywords:** exocytosis, super-resolution microscopy, palmitic acids, STED, storm, SNARE proteins, membrane fusion

## Abstract

Exocytosis, the process in which material is transported from the cell interior to the extracellular space, proceeds through a complex mechanism. Defects in this process are linked to a number of serious illnesses including diabetes, cancer, and a range of neuropathologies. In neuroendocrine cells, exocytosis involves the fusion of secretory vesicles, carrying signaling molecules, with the plasma membrane through the coordinated interplay of proteins, lipids, and small molecules. This process is highly regulated and occurs in a complex three-dimensional environment within the cell precisely coupled to the stimulus. The study of exocytosis poses significant challenges, involving rapidly changing, nano-scale, protein–protein, and protein–lipid interactions, at specialized sites in the cell. Over the last decade our understanding of neuroendocrine exocytosis has been greatly enhanced by developments in fluorescence microscopy. Modern microscopy encompasses a toolbox of advanced techniques, pushing the limits of sensitivity and resolution, to probe different properties of exocytosis. In more recent years, the development of super-resolution microscopy techniques, side-stepping the limits of optical resolution imposed by the physical properties of light, have started to provide an unparalleled view of exocytosis. In this review we will discuss how advances in fluorescence microscopy are shedding light on the spatial and temporal organization of the exocytotic machinery.

## Introduction

Regulated exocytosis is a fundamental process of multicellular life, permitting cells with diverse functions, and properties to act in a coordinated manner. In vertebrates, this cell–cell communication occurs over short distances and between small numbers of cells, for example in neurons, or over longer ranges encompassing many cell types and organs in the case of neuroendocrine signaling (1). In all examples of regulated exocytosis, the final stages of membrane fusion, lipid mixing, and content release are catalyzed by the coordinated action of the SNARE (soluble NSF attachment protein receptor) proteins (2–4). In neurons and neuroendocrine cells three SNARE proteins act synergistically to drive membrane fusion; syntaxin and SNAP-25 on the plasma membrane, and synaptobrevin in secretory vesicles (4–6). Together, these three proteins form a four helical complex, which drives the merger of the plasma and vesicular membranes (7–9). *In vitro* biochemical studies have described many of the individual stages of SNARE protein action; through the formation of a stable binary intermediate, the zippering of the SNARE complex, the formation of cis and trans complexes, and established the SNAREs as the minimal machinery required for exocytosis (6, 8–13). In parallel, *in vivo* studies have demonstrated the importance of the SNARE proteins in the physiological development and functioning of secretory cells and organs (14–16).

**Figure 1:**
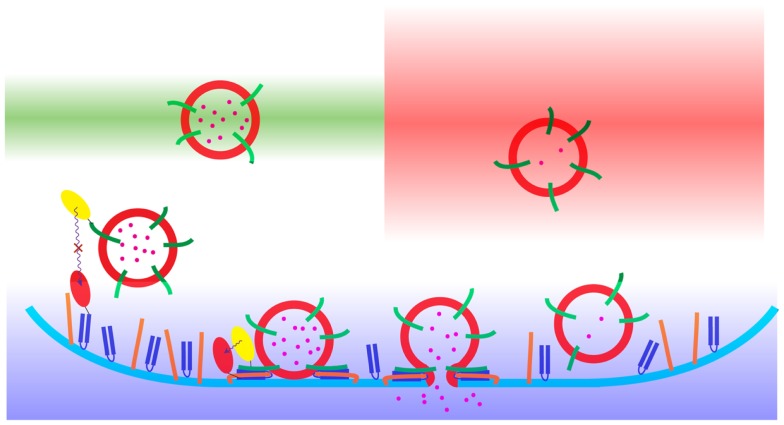
**A schematic representation of the process of neuroendocrine exocytosis as observed using diffraction-limited fluorescence microscopy**. The synaptic vesicles (red) with the cargo (pink) undergo fusion with the plasma membrane (blue). The color gradients show the areas of the sample acquisition: CLSM (green), WF (red), and TIRFM (blue). The principle of Förster resonance energy transfer (FRET) microscopy is presented with the use of yellow ovals [located on synaptobrevin (green)] and red ovals [located on SNAP-25 (navy blue)]. The Förster resonance energy transfer occurs only when the distance between the donor (yellow) and the acceptor (red) fluorophores are in the range of 1–10 nm. The third SNARE protein, syntaxin, is denoted in orange.

**Figure 2:**
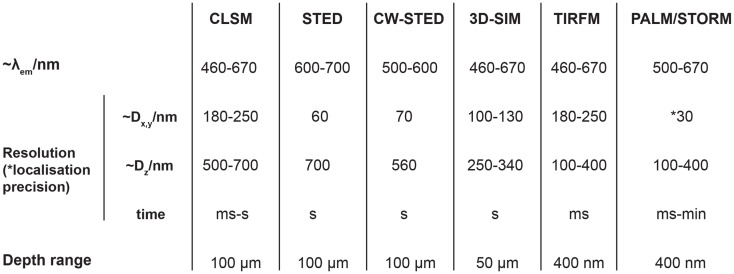
**Summary of diffraction-limited and super-resolution microscopy approaches**. Seven microscopy techniques are detailed: confocal laser scanning microscopy (CLSM), stimulated emission depletion microscopy (STED), continuous wave STED (CW-STED), structured illumination microscopy (3D-SIM), total internal reflection fluorescence microscopy (TIRF), photoactivation localization microscopy (PALM), and stochastic optical reconstruction microscopy (STORM). The table shows the typical operational emission wavelengths, lateral and axial resolutions, the temporal resolution of data acquisition, and the sampling depth range. For PALM and STORM microscopy the typical lateral localization precision is stated. For further information regarding the referenced techniques (63).

This complex cascade of protein–protein interactions provides challenges and opportunities to investigation through the use of optical microscopy (Figure 1). Optical microscopy inherently provides spatial information, and with improvements in detection technologies, enhanced sensitivity, and speed. By sampling specific wavelengths of visible light, in fluorescence microscopy, it is possible to discriminate multiple labels within a sample (17). This has been used extensively to study exocytotic events in a large number of secretory cell types from dissociated single cells up to intra-vital imaging in whole organisms. The use of fluorescence microscopy provides a huge improvement over electron microscopy, which is incompatible with live cell imaging and often suffers from an inability to discriminate between objects in the resulting image. However, fluorescence microscopy achieves this at a cost; significantly lower resolution. While electron microscopy is ultimately able to resolve objects to the sub-nanometer range, permitting the generation of protein structures with atomic resolution, fluorescence microscopy is typically limited in resolving power to hundreds of nanometers. The resolution on an optical microscope is limited by two factors, aberration and diffraction, which both serve to blur the image and limit the ability to distinguish two adjacent points (17). Aberration can be corrected through the expensive optics found on modern microscopes, but diffraction is ultimately governed by the wavelength of light and the aperture of the objective according to the Abbe formula and Rayleigh criterion (17, 18). Most fluorophores used in cellular imaging emit light in the wavelength range of 400–700 nm which results in a lateral limit of resolution for standard diffraction-limited microscopes of between 170 and 300 nm respectively (Figure 2). Now, new technological breakthroughs are pushing the limits in resolution in the spatial and temporal domains revising our understanding of the process of neuroendocrine exocytosis. This short review will cover some of the highlights of how microscopy has advanced our understanding of neuroendocrine secretion.

## Temporal Investigation of the Process of Exocytosis

A key characteristic of all regulated exocytosis, regardless of the type of specialized cell studied is that it is a rapid process. Globally, for all secretory vesicles in a cell the rate of cargo release may be slow and multiphasic, but at the single vesicle level the process of bilayer merger and cargo release occurs over a very fast timescale (19, 20). To examine a dynamic process using fluorescence microscopy requires a high temporal resolution. This is difficult to achieve using confocal laser scanning microscopy, due to the requirement for scanning (Figure 2). Widefield techniques, which provide spatial information through sensitive cameras can achieve high temporal sampling frequencies at the expense of axial resolution (17). A major advance was achieved through the invention of the total internal reflection fluorescence microscope (TIRFM), which uses total internal reflection to establish an evanescent wave at the coverslip (21). This permits the high temporal resolution of widefield camera-based microscopy but in a thin axial section at the plasma membrane. One of the first applications of this technique was to observe the fusion of secretory vesicles with the plasma membrane (19, 22, 23). Initial experiments examining the fusion process utilized acidic dyes, such as Acridine orange, but these were soon superseded by fluorescently labeled cargo molecules (24, 25). In these early studies it was clear that, at rest, the secretory vesicles are highly dynamic in the cell interior while vesicles at the plasma membrane were largely immobile (22, 26, 27). This supported the hypothesis from electrophysiological data that different pools of secretory vesicles exist with different release properties (20). Secretory vesicles at the plasma membrane do not exhibit free diffusion but instead demonstrate a caged, or tethered behavior, termed morphological docking. Upon stimulation a proportion of these morphologically docked vesicles fuse with the plasma membrane releasing their cargo (28). Using high speed imaging under TIRFM illumination, Degtyar and colleagues demonstrated that immediately prior to fusion, secretory vesicles undergo a rapid lateral movement (29). The mechanisms behind this are unclear but it may serve to sample a larger area of the plasma membrane, enhancing the probability of SNARE interactions, or be a direct result of the formation of the SNARE complexes themselves. While TIRFM provides high temporal resolution of exocytotic events at the plasma membrane, it cannot observe events over the entire cell surface or at depth, in clusters of cells (17). Spinning disk confocal microscopy has been employed to study vesicle fusion throughout cells clusters and in polarized pancreatic acini (30, 31). These studies are beginning to move the temporal study of vesicle dynamics and fusion from single cells toward whole organs and beyond.

While secretory vesicles provide an ideal object to image, being larger and more sparsely distributed than the limits imposed by diffraction, they are only one part of the story of exocytosis. Imaging of proteins is confounded by limited resolution, however, this has not prevented the development of innovative solutions to probe SNARE protein function with high temporal resolution. Fluorescence recovery after photobleaching (FRAP) has been widely used to examine the molecular motion of SNARE proteins on the plasma membrane (32). By selectively bleaching fluorescently labeled SNAREs in a sub region of the plasma membrane the recovery of fluorescence in this region through diffusion can be monitored. Sieber and co-workers used FRAP to measure the diffusion of syntaxin molecules on the plasma membrane to derive a dynamic model of SNARE organization (33). In a subsequent study by the same group FRAP was used to probe the intermolecular interactions of SNAP-25 and syntaxin (34), supporting *in vitro* observations of a 1:1 binary intermediate (11, 13). FRAP provides a global average for the diffusion rate of the population of molecules being studied. Due to the high density of the molecules, their individual motion cannot be resolved using diffraction-limited microscopy. However, using an adaptation of a scanning confocal microscope in which the laser beam is parked to a single point in space, diffusion of molecules through the beam can be monitored. This approach called fluorescence correlation spectroscopy (FCS) has been used extensively in *in vitro* reconstituted studies of SNARE protein organization (35–37). FCS studies showed that synaptobrevin and syntaxin both preferentially sequester in the liquid-disordered phase of giant unilamellar vesicles (35). This argues against a classical raft hypothesis for SNARE protein organization, however, the situation in cellular membranes may be more complicated due to the diversity of lipid species present.

## Resolving the Molecular Organization and Interactions Underlying Exocytosis

The proteins responsible for driving the process of exocytosis are un-resolvable due to their size and high density in neuroendocrine cells using diffraction-limited microscopes. In the last 5 years there has been a revolution in cell imaging with the introduction of super-resolution microscope techniques, which have pushed the ability to resolve objects through supramolecular imaging down to single molecule detection (38) (Figure 2). These techniques can be broadly divided into two classes; hardware approaches and localization approaches. Stimulated emission depletion microscopy (STED) and structured illumination microscopy (SIM) achieve the enhancement in resolution through alterations in the illumination of the sample (39–41). This achieves a two to sixfold enhancement in the optical resolution of the microscope over the theoretical limit imposed by the diffraction of light. STED can be implemented using either pulsed lasers or constant wave lasers (CW-STED) (42); the former providing enhanced lateral resolution while the latter provides simpler implementation. The second class of super-resolution techniques utilize localization of emission to achieve single molecule imaging (43). These single molecule localization microscopy (SMLM) techniques include photoactivation localization microscopy (PALM), ground state depletion with individual molecule return (GSDIM), and stochastic optical reconstruction microscopy (STORM and dSTORM) (44–47). All of these SMLM approaches take advantage of the ability to switch molecules between light and dark states to decrease the number of fluorescent molecules observed at one time. By repeated recording it is then possible to observe many tens of thousands of individual, fluorescently labeled, proteins and compute their position with a precision of 5–20 nm.

These super-resolution techniques have been utilized to investigate the organization of the plasma membrane secretory machinery. Over the last decade it has become clear that the plasma membrane SNAREs, syntaxin, and SNAP-25 are not uniformly distributed over the plasma membrane, but instead are observed to exists in a clustered morphology (13, 48–53). These studies used diffraction-limited techniques and largely agreed that clusters were of the order of 200 nm in diameter. It is important to note that using these microscopes, as a result of diffraction of the light through the optics, any object of approximately 200 nm or less would appear this size. Regardless, a number of important observations were made with relation to the dependency of the integrity of these clusters on cholesterol and their partial colocalization with secretory vesicles (48–50). In 2007 a major advance was reported using STED microscopy, which showed that syntaxin forms clusters of approximately 50 nm in size. Based on computer modeling this supramolecular assembly was hypothesized to contain around 70 molecules of syntaxin and required functional SNARE domains of syntaxin (33). In a subsequent study STED was used to probe the involvement of phosphatidylinositol (4,5) bisphosphate (PIP2) in syntaxin clustering. This study concluded that cluster formation required the combined clustering of PIP2 and syntaxin acting synergistically together (54). Importantly, the measured size of the clusters falls at the limit of accuracy of the STED microscope used and so this can only act as an upper limit to the size of a cluster. More recently, SMLM has been employed to investigate SNARE organization on the plasma membrane (55, 56). This goes beyond the observation of multi-protein structures to localize the individual molecular components. dSTORM imaging demonstrated a clustered morphology for both syntaxin and SNAP-25 (55, 56). This non-homogeneous distribution was also observed using PALM and recapitulated in live cells using single particle tracking PALM (sptPALM) (56). This latter technique also observed that syntaxin and SNAP-25 molecules cannot freely diffuse on the plasma membrane, but instead are restricted to as yet undefined microdomains. Interestingly using these super-resolution molecular techniques, the secretory vesicles were not localized to the higher density plasma membrane SNARE clusters (56). This appears at odds with the previous observation of partial colocalization with SNARE clusters using diffraction-limited techniques (48–50). However, this apparent discrepancy is most likely due to the level of “detail” resolved using the respective technique.

Although super-resolution techniques provide unparalleled resolution they are limited to describing the position of protein molecules with no information on interaction status. However, it is possible to probe interactions of molecules over distances below 10 nm using Förster resonance energy transfer (FRET) microscopy (57). This has been employed to study protein–protein interactions between the SNAREs, providing key insights into how these molecules interact in a cellular environment and how this is regulated (54, 58–60). There are two main approaches for the determination of FRET; intensity based techniques including sensitized emission and acceptor photobleaching, and fluorescence lifetime imaging (FLIM) (17). All of these techniques measure the effect of non-radioactive energy transfer between the donor and acceptor molecules. In terms of SNARE clustering, FRET has successfully been used to examine protein clustering in artificial liposomes, observing clustering through exclusion from high cholesterol regions as well as probing the intra-cluster interactions of syntaxin and SNAP-25 (52, 61, 62).

## Conclusion

The development of new microscopy techniques with ever higher temporal and spatial resolution has mirrored the requirements and advancements of the field of neuroendocrine secretion. This biological system serves as an excellent test bed for many of the new emerging technology as a result of our already considerable understanding and also the specific problems and opportunities offered. The next big step is to combine super-resolution, high speed, and functional imaging into single experiments. The iterative process of technological advancement and new biological findings will continue until the ultimate experiment of being able to watch a complete single fusion event with sub-molecular resolution of multiple protein components is achieved.

## Conflict of Interest Statement

The authors declare that the research was conducted in the absence of any commercial or financial relationships that could be construed as a potential conflict of interest.
